# Category-Based Effect on False Memory of People with Down Syndrome

**DOI:** 10.3390/brainsci14060538

**Published:** 2024-05-24

**Authors:** Ching-Fen Hsu, Qian Jiang, Shi-Yu Rao

**Affiliations:** 1 School of Foreign Languages, Hunan University, Changsha 410082, China; 2 Laboratory for Language Pathology and Developmental Neurosciences, Hunan University, Changsha 410082, China

**Keywords:** Down syndrome, false memory, concept formation, nouns, verbs

## Abstract

**Background**: People with Down syndrome (DS) are deficient in verbal memory but relatively preserved in visuospatial perception. Verbal memories are related to semantic knowledge. Receptive ability is better than expressive ability in people with DS but still seriously lags behind their age-matched controls. This lag may result in the weak semantic integration of people with DS. **Aims**: This study aimed to examine the ability of semantic integration of people with DS by using false-memory tasks. Possible differences in the number of false memories induced by nouns and verbs were of focus. **Methods and Procedures:** Two phases were involved in the false-memory task. In the study phase, ten-word lists with semantically related associates were presented. In the recognition phase, judgments were to be made about whether the words presented had been heard before. Three types of words were tested: previously presented associates, semantically related lures, and semantically unrelated new words. **Outcomes and Results**: People with DS overall showed the lowest accuracy among groups in response to tested word types. In the processing of lures, people with DS were worse in recognition than MA controls. In processing unrelated words, people with DS responded least accurately to all types of words compared to control groups. In the processing of associates, people with DS showed similar recognition rates as the MA controls but were less accurate than the CA controls. No difference was observed between nouns and verbs in recognizing word types among groups, though faster responses to nouns than to verbs emerged in college students. Further analyses on topic-wised comparisons of errors across syntactic categories revealed differences in specific concepts among groups, suggesting people with DS were atypical in semantic organization. **Conclusions and Implications**: People with DS showed mixed patterns in semantic integration by false-memory tasks with delay to associates and deviance to lures together with unrelated words. People with DS showed distinct patterns in processing nouns and verbs while conducting topic-wise comparisons, suggesting that they formed false memories differently based on distinct syntactic categories. We concluded that people with DS develop a deviant semantic structure, hence showing problems in language and social cognition. Category-based rehabilitation is suggested to be implemented for people with DS to improve their semantic knowledge through lexical connections.

## 1. Introduction

People with Down syndrome (DS) have a genetic disorder of chromosome 21, with common trisomy of the upper or lower arm. The reported etiology constitutes approximately 1 in 700 live births according to the Centers for Disease Control and Prevention in 2024, and it is one of the most severe congenital disorders according to the World Health Organization in 2023. Due to this genetic deficit, people with DS are individuals with intellectual disorders and have abnormal cognitive ability with poor language but relatively good visuospatial ability [1]. People with DS have protruded tongues, slanted eyelids, short limbs, and short trunks [2]. Furthermore, people with DS present typically developed implicit memory and explicit memory impairment [3]. According to Roediger and McDermott [4], false memory is to recall or recognize events that are not true or do not exist. Participants listened to word lists that were semantically related, forming gist themes in their mental representation. However, participants did not realize the concept formation while listening to the word lists in the study phase. Later in the recognition phase, participants were asked to make judgments about whether each shown word had been presented before. The ability of concept formation is very important in discourse comprehension and serves as the foundation of social cognition. Given poor knowledge of lexical semantics but better receptive abilities in people with DS [5], it was of interest to find to what extent people with DS integrated lexical words into sentences compared to the typically developing (TD) controls. That is, the way by which people with DS integrate semantically related words into coherent concepts remains unknown. This was the main focus of this study.

Deviant semantic knowledge in processing ambiguous words in contexts was observed in people with DS [6]. People with DS showed the lowest accuracy in integrating ambiguities into preceding contexts, suggesting less capability in selecting the correct interpretation of ambiguities. In grouping semantic-related concepts [7], people with DS showed deviancy in priming picture backgrounds (e.g., a sports store) and picture objects (e.g., a pair of sneakers vs. a pair of high heels). While people with Williams syndrome (WS) and the mental age (MA) controls primed only the semantic-related pairs, people with DS primed both semantic-related and -unrelated pairs. This finding indicates that people with DS have coarser semantic organization compared to the controls. Hsu [7] examined the comprehension of figurative words embedded in contexts in people with DS, testing both forward and backward reasoning. The first was examined through clauses expressing cause and consequence in this order, such as “Sponge Bob would like to eat the candies on a shelf. He asked for help from Squidward Tentacles, but Squidward Tentacles dampened Sponge Bob’s enthusiasm” (original Chinese text: 海绵宝宝想吃柜子上的糖果, 找章鱼哥帮忙, 结果被章鱼哥泼冷水). The last three characters formed a phrase with the figurative meaning “to dampen one’s enthusiasm” and the literal meaning “to pour cold water on someone”. Backward reasoning was examined through linked clauses expressing consequences resulting from a cause (in this order); for example, “Daxung failed the exam this time. His mother often reminded him to study hard, but Daxung was inattentive to his mother’s reminders” (original Chinese text: 大雄这次考试不及格, 妈妈平常叫大雄认真读书, 大雄都把妈妈的话当作耳边风). The sentences could only be comprehended when the participants understood the figurative meaning of the words. The results revealed that people with DS were deviant in processing backward reasoning and delayed in processing forward reasoning through comprehending ambiguities at sentential final positions. This finding suggested that people with DS are quite poor at lexical semantics.

Syntactic categories were reported to develop asymmetrically in people with DS [8]. In Loveall et al.’s study [8], people with DS aged 4–7.11 years old were observed to be delayed in verbs compared to nouns and adjectives in a study with the Peabody Picture Vocabulary Test (Fourth Edition) [9]. The results demonstrated deficient lexical knowledge of verbs in people with DS. While people with DS showed a similar extent of accuracy in terms of adjectives and nouns, MA controls showed better accuracy for nouns than adjectives. This finding confirmed the delayed development of verbs and distinct processing of adjectives in people with DS. Another study on inferential language use through storytelling provided further evidence of the impairment of syntactic categories in people with DS [10]. In the study of Ashby et al. [10], the subtype of inferences used that are embedded within character actions or attempts in the story was tested, such as (1) prediction of actions [e.g., the frog *is going to jump* into the salad], (2) emotional behaviors [e.g., she *yells* at the frog], (3) goal-achieving actions [e.g., he *tried* to catch the frog], and (4) descriptions of actions [e.g., the frog *jumped* in the pocket]); the results revealed that people with DS were worse than TD controls. A similar pattern was observed in another subtype of inferences used that are embedded within the internal state, which refers to (1) character thought or belief inference [e.g., she *noticed* the frog in the salad], (2) character preferences or needs [e.g., he *wants* to catch the frog], (3) character perceptions [e.g., he *sees* the frog in the saxophone], (4) reference to character personality [e.g., the *nice* woman], and (5) emotion states [e.g., the waiter was *mad*]); the results revealed that people with DS were worse than TD controls. In other subtypes of inference use in (1) negation [e.g., he *did not* know where the frog was], (2) location [e.g., he drove us *home*], (3) possession [e.g., the frog got in *his* pocket], and (4) role [e.g., *Dad* paid the man]), people with DS were worse than people with fragile X syndrome, even when the MLU was matched. Moreover, people with DS and the TD controls at their MA level showed no significant differences. These findings suggested that people with DS are impaired in lexical knowledge of verbs, nouns, and adjectives. Put together, these deficits all result in difficulties of comprehension in people with DS. However, it is still unclear how people with DS integrate semantically related words into coherent concepts given their poor knowledge of lexical semantics. The aim was to examine how people with DS processed lexical words with different semantic linkage weights. The processing pattern would reveal the semantic knowledge of people with DS by investigating their ability of concept formation. A minor aim was to examine whether the concept integration in semantic knowledge would be influenced by syntactic categories, namely, nouns and verbs, in people with DS. It was of interest to examine whether people with DS would show the same developmental delay as their mental age-matched controls or developmental deviance which was not similar to any control group. That people with DS would show bizarre knowledge of lexical semantics and developmental deviance in concept formation with more difficulty in verbs was hypothesized. To answer these questions, false-memory tasks were conducted to measure semantic weights from themes to associates and unrelated words across nouns and verbs. The discrimination sensitivities and errors of word types were analyzed to reveal the semantic knowledge of each group.

## 2. Method

### 2.1. Participants

Twenty people with DS diagnosed with trisomy 21 in hospitals at various ages were recruited. Twenty typically developing (TD) controls were individually matched with people with DS based on chronological age (CA) and mental age (MA) using the Wechsler Scale of Intelligence for Children (WSIC, Chinese version, China). The matching criteria for the TD controls were within the range of 6 months older or younger than those with DS. All participants were right-handed native speakers of the Chinese language. The mean age of CA in people with DS and CA-matched controls was 17.7 years old; the mean age of MA in people with DS and MA-matched controls was 9.4 years old. Age variations yielded no significant differences between people with DS and CA- or MA-matched controls in the comparisons of corresponding age (see Table 1).

Twenty college students were recruited to ensure the validity of the materials that could form the gist concepts. Another 40 college students participated in generating noun and verb stimuli (20 each). Another 40 college students participated in rating the noun and verb stimuli (20 each). All college students were recruited from Hunan University, Changsha, China. The elementary students and junior high students were recruited from Primary School Affiliated with Hunan University and Dongkou Wenchang Street Second Primary School in Changsha. People with DS were recruited from the Association for Parents of Down Syndrome in Changsha and Leyi Integrated Special Education Institution in Chongqing. This study was approved by the Institutional Review Board of the School of Foreign Languages at Hunan University (20210628000004).

### 2.2. Materials and Design

Roediger and McDermott’s [4] classic false-memory paradigm was used as the research method. Twenty lists of nouns and verbs (ten each) were generated. Two tasks were conducted to have nouns and verbs tested in consecutively separate tasks. Each participant completed two phases: study and recognition. In the study phase, ten semantically related associates were randomly presented to the participants. In the recognition phase, three types of words were presented consecutively for participants to judge whether they had heard the words in the study phase: (1) semantically related nonpresented lures, (2) previously presented associates, and (3) semantically unrelated nonpresented new words. Hence, each participant listened to 100 words in the study phase and 90 words in the recognition phase in each task. Among the 90 words tested in the recognition phase, 30 associates, 30 semantically unrelated words, and 30 semantically related lures were presented.

### 2.3. Word-Generating Task

Words were generated to obtain the stimuli of nouns and verbs. Eighty-four target nouns were collected by the experimenters from cartoons, comic books, the Baidu search engine, and frequent scenarios familiar to children, such as *amusement park*, *bathroom*, and *barbershop.* These target nouns were put into the word-generating task later. Twenty college students (10F/10M, mean CA = 22.6, SD = 0.6, age range = 18.7–27.9) were asked to write down as many associates as possible related to each target noun, without a time limit. All the generated associates were collected. The frequency ratio was calculated by dividing the total frequency of all associates by the frequency of the top 12 associates. The top 12 nouns with the highest frequency ratio were chosen as the target nouns. The associates of each target noun were selected as words presented in the study phase of the false-memory task. Since the generated associates could be shown to be synonyms which were semantically related in the same semantic field, the frequency of the same semantic field was cumulated (e.g., bing1-ji1-ling2 [冰激凌] *ice cream* and bing1-bang2 [冰棒] *ice stick*). The generated associates of each target noun were not restricted to specific categories, all selected associates were high-frequency concepts and familiar to participants.

In the process of selecting verbs, eighty-five verbs underwent a word-generating task from cartoons, comic books, the Baidu search engine, and activities that children are familiar with, e.g., *doing housework* or *celebrating birthdays*. Another 20 college students (10F/10M, mean CA = 21.4, SD = 3.5, age range = 18.1–31.1) were recruited to write down as many associates as possible related to each verb, without a time limit. The top 12 verbs with the highest frequency ratio were chosen as the target verbs. The associates of each target verb were selected as words presented in the study phase of the false-memory task. Since the generated associates could be shown to be synonyms that were semantically related in the same semantic field, the frequency of the same semantic field was cumulated (e.g., 洗衣服 [xi3-yi1-fu2] and 洗衣 [xi3-yi1] both mean *doing laundry*). The generated associates of each target verb were not restricted to specific categories. Though the generated words were not specified in syntactic categories, the associates were semantically related in concepts with high frequency among one another. The semantic links of associates of each topic were elicited. This was the key feature of the stimuli that were controlled.

### 2.4. Word-Rating Task

A word-rating task was conducted to determine the semantic closeness of the target syntactic categories and associates. For each syntactic category, the top two most strongly semantically related associates to the selected topic were taken as lures. The remaining 10 semantically related associates and the three semantically unrelated words were rated by college students (20 for nouns, 20 for verbs).

A total of 150 pairs of noun stimuli, including 20 pairs of lures, 100 pairs of associates, and 30 pairs of unrelated words, were judged from the highest, 5, to lowest, 1, based on the semantic relatedness of the pairs. For example, 医院–医生 (*hospital*–*doctor*) received the highest rating of 5 because of the strongest semantic relatedness, while 家具店–零食 (*furniture store*–*snack*) received the lowest rating of 1 due to semantic irrelevance. The results revealed high semantic relatedness among three types of words: 4.78 (SD = 0.43) for lures, 4.28 (SD = 0.57) for associates, and 1.29 (SD = 0.61) for unrelated words [lures vs. associates, *t*(199) = 13.75, *p* < 0.001; lures vs. unrelated words, *t*(199) = 64.30, *p* < 0.001; associates vs. unrelated words, *t*(199) = 52.86, *p* < 0.001].

Another 150 pairs of verb stimuli, including 20 pairs of lures, 100 pairs of associates, and 30 pairs of unrelated words, were judged from highest, 5, to lowest, 1, based on the semantic relatedness of word pairs. The rating results revealed significant differences in semantic relatedness in verbs: 4.31 (SD = 0.67) for lures, 3.87 (SD = 0.61) for associates, and 1.38 (SD = 0.64) for unrelated words [lures vs. associates, *t*(199) = 9.56, *p* < 0.001; lures vs. unrelated words, *t*(199) = 43.19, *p* < 0.001; associates vs. unrelated words, *t*(199) = 41.00, *p* < 0.001].

The noun stimuli were recorded at 44.1 kHz with Praat sound-editing software (https://www.fon.hum.uva.nl/praat/download_win.html). The average auditory length was 0.88 s (SD = 0.22 s, range = 0.60–1.54; mean length of characters = 2.50, SD = 0.63) for lures, 0.84 s (SD = 0.15 s, range = 0.58–1.27; mean length of characters = 2.22, SD = 0.44) for associates, and 0.81 s (SD = 0.08 s, range = 0.70–1.05; mean length of characters = 2.00, SD = 0.00) for unrelated words. One-way ANOVA analyses with the same auditory length as the within-participants factor and the same word type as the between-participants factor yielded no differences in auditory length across word types. However, the auditory length based on topics in word lists, including lures, associates, and unrelated words, yielded a significant difference [*F*(9,150) = 2.818, *p* = 0.004, *ƞ*^2^ = 0.145]. The results revealed that some word lists (e.g., under the topics of *breakfast store* [0.784, SD = 0.082], *public dancing* [0.802, SD = 0.100], *bathroom* [0.744, SD = 0.093], *hospital* [0.804, SD = 0.131]) were responded to less accurately than others (e.g., under the topics of *amusement park* [0.933, SD = 0.234], *road* [0.915, SD = 0.150], *Christmas* [0.905, SD = 0.215]). Character lengths of the word types were significant [*F*(2,157) = 9.688, *p* < 0.001, *ƞ*^2^ = 0.110], suggesting all three types of words were indeed distinct from one another (lures, 2.5, SE = 0.81 vs. associates, 2.22, SE = 0.44, *p* = 0.003; associates vs. unrelated, 2, SE = 0.081, *p* = 0.003; lures vs. unrelated, *p* < 0.001).

The verb stimuli were recorded at 44.1 kHz with Praat sound-editing software. The average auditory length was 0.88 s (SD = 0.15 s, range = 0.72–1.29; mean length of characters = 2.13, SD = 0.35) for lures, 0.88 s (SD = 0.20 s, range = 0.63–1.40; mean length of characters = 2.18, SD = 0.39) for associates, and 0.85 s (SD = 0.12 s, range = 0.66–1.38; mean length of characters = 2.03, SD = 0.18) for unrelated words. The analyses of ANOVA with repeated measures of the auditory length of word types as a within-participants factor revealed that no difference was observed across the types of words or word lists for verbs. Auditory length based on verb topics yielded no differences. The character lengths of the word types of verbs were not significant.

The noun stimuli were recorded by a male (CA = 26.9) and the verb stimuli were recorded by a female (CA = 25.3). Since this was a within-participant design, the stimuli across distinct syntactic categories were recorded by a male and a female to make the difference salient and keep the participants’ attention.

### 2.5. Procedures

There were two phases in the false-memory task. In the study phase, a fixation point was displayed on the computer screen for 1000 ms. Word lists were presented aurally one by one via speakers, with an average presentation rate of 2000 ms for each word in a clear voice. The volume was adjusted to ensure comfort for each participant. The interstimulus interval was 100 ms.

In the recognition phase, a 500 ms fixation point was followed by a test word. The presentation rate was the same, at 2000 ms for each word in a clear voice. The participants had to make a yes-or-no judgment as to whether the word had been heard previously in the study list by pressing the corresponding button. Buttons *d* and *k* were counterbalanced to indicate positive or negative responses with stickers on the keyboard. The stickers in green and red served as reminders for positive and negative responses, with two circles on the green square and one cross with one circle on the red square on the computer screen. So, no confusion was involved in button pressing, which was counterbalanced in responses for participants. Before the experiment began, participants were given time to practice. There was no time limit for the responses. Participants received the study phase and the recognition phase accordingly. The noun-eliciting false-memory task and the verb-eliciting false-memory task lasted about 10 min. No break was in between. Reaction times and response accuracies were measured. Each participant received the study in a quiet room.

The instruction was as follows: “Nice to meet you! You are playing a computer game now. There are two parts to this game. In the first part, the computer will speak a series of words to you one by one continually via speakers. You have to pay full attention to it. In the second part, the computer will speak one word at a time via speakers. Upon hearing a word, you decide whether you have heard the word in the study phase before. If yes, press the green button; if no, press the red button. As long as you know the answer, you should press the button right away. Are you ready to play the game? Let’s start!”

## 3. Results

The rationale for data analyses is listed below. Two within-participants factors, i.e., syntactic category and word type, and one between-participants factor, i.e., group, were put into a three-way ANOVA. First, we analyzed the data from college students to find out the typical pattern and possible impact of syntactic categories in false memory. Later, we analyzed the data from the DS group and the TD controls. Third, the discrimination sensitivities between associates from lures and associates from unrelated words were calculated in the noun- and verb-eliciting false-memory tasks. Fourth, error patterns in the noun- and verb-eliciting false-memory tasks were analyzed.

### 3.1. Analyses of College Data

**Figure 1:**
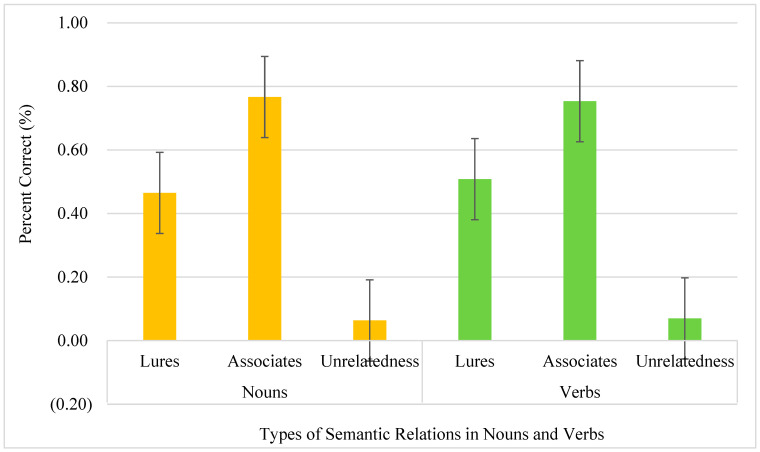
Accuracy of types of semantic relations in nouns and verbs of college students.

Twenty college students (10F/10M, CA = 23.5, SD = 2.4, range = 18.8–26.11, SD = 2.4) took part in this study. The data of college students served as the baseline for later comparisons of people with DS and the TD control groups. “Yes” responses were included in the analyses. The types of words and syntactic categories were taken as the within-participants factors in repeated measure analyses of variance. In the analysis of the “yes” responses, no interactions emerged (*F* < 1). The main effect of types of words was significant [*F*(2,38) = 138.56, *p* < 0.001, *ƞ*^2^ = 0.879], suggesting the lowest “yes” responses to unrelated words (0.07, SE = 0.024) and highest “yes” responses to associates, i.e., previously presented old words (0.76, SE = 0.030). The proportion of “yes” responses to lures (0.49, SE = 0.037) was in between. No difference was observed in the syntactic category (verbs, 0.44, SE = 0.022; nouns, 0.43, SE = 0.019). The accuracy of each type of semantic relationship with error bars is shown in Figure 1.

**Figure 2:**
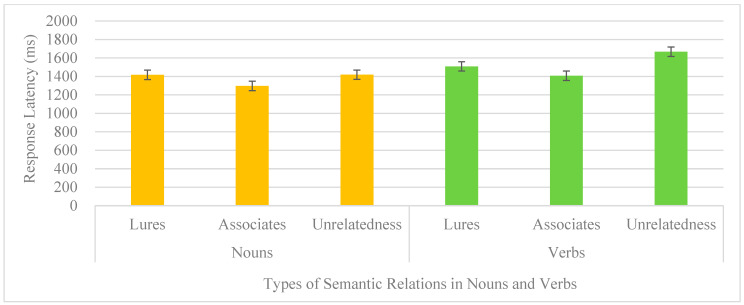
Response latency of types of semantic relations in nouns and verbs of college students.

Follow-up studies on response latency, types of words, and syntactic categories were considered within the participants with repeated measures. No interaction was observed (*F* < 1). The main effect of the syntactic category was significant [*F*(1,9) = 6.92, *p* = 0.027, *ƞ*^2^ = 0.43], suggesting nouns (1377, SE = 57) were responded to faster than verbs (1527, SE = 80). The main effect of types of words was also significant [*F*(2,18) = 5.49, *p* = 0.014, *ƞ*^2^ = 0.37], implying associates were responded to fastest (1352, SE = 37) and unrelated words were responded to slowest (1542, SE = 99), with lures in between (1463, SE = 65). The response latencies for each type of semantic relations in nouns and verbs with error bars are shown in Figure 2. The patterns shown by college students in responding to nouns and verbs served as the baseline for later comparisons of the clinical group and their age-matched control groups.

### 3.2. Data Analyses of People with Down Syndrome and Typically Developing Controls

No three-way interactions were observed (*F* < 1). Two-way interaction between types of words and groups was significant [*F*(4,114) = 12.06, *p* < 0.001, *ƞ*^2^ = 0.29]. One of the simple main effects was from the difference among groups in each type of word, suggesting group difference in lures [DS, 0.518, SE = 0.040; CA, 0.441, SE = 0.040; MA, 0.359, SE = 0.040; *F*(2,57) = 3.96, *p* = 0.025, *ƞ*^2^ = 0.122], associates [CA, 0.709, SE = 0.031; MA, 0.592, SE = 0.031; DS, 0.588, SE = 0.031; *F*(2,57) = 4.931, *p* = 0.011, *ƞ*^2^ = 0.147], and unrelated words [DS, 0.355, SE = 0.032; MA, 0.157, SE = 0.032; CA, 0.101, SE = 0.032; *F*(2,57) = 17.270, *p* < 0.001, *ƞ*^2^ = 0.377]. The difference in lures was due to the difference between the DS and MA (*p* = 0.007). The difference in associations was due to the difference between DS and CA (*p* = 0.008) and the difference between MA and CA (*p* = 0.01). The difference in unrelated words was due to the difference between DS and CA (*p* < 0.001) and DS and MA (*p* < 0.001). Another simple main effect was the difference in the types of words used in each group. In the CA group, associates were responded to more accurately (0.709, SD = 0.157) than lures (0.440, SD = 0.153) and unrelated words (0.100, SD = 0.196) at *p* < 0.001 [*F*(2,38) = 91.743, *p* < 0.001, *ƞ*^2^ = 0.828]. The latter two types of words differed significantly (*p* < 0.001). In the MA group, associates were responded to more accurately (0.591, SD = 0.123) than lures (0.359, SD = 0.131) and unrelated words (0.156, SD = 0.132) at *p* < 0.001 [*F*(2,38) = 77.022, *p* < 0.001, *ƞ*^2^ = 0.802]. The latter two types of words differed significantly (*p* < 0.001). In the DS group, associates were responded to more accurately (0.588, SD = 0.132) than lures (0.517, SD = 0.232) and unrelated words (0.355, SD = 0.187) at *p* < 0.001 [*F*(2,38) = 22.058, *p* < 0.001, *ƞ*^2^ = 0.537]. The latter two types of words differed significantly (*p* < 0.001). The difference between associates and lures was not significant.

Two-way interaction of syntactic categories and types of words reached significance [*F*(2,114) = 4.718, *p* = 0.011, *ƞ*^2^ = 0.076]. The simple main effect was from the difference among types of nouns [*F*(2,118) = 97.864, *p* < 0.001, *ƞ*^2^ = 0.624] and verbs [*F*(2,118) = 131.211, *p* < 0.001, *ƞ*^2^ = 0.690]. For nouns, associates (0.619, SE = 0.021) received higher responses than lures (0.441, SE = 0.027) or unrelated words (0.224, SE = 0.025) [all *p* < 0.001]. For verbs, associated words (0.641, SE = 0.022) received higher responses than lures (0.438, SE = 0.024) or unrelated words (0.184, SE = 0.024) [all *p* < 0.001]. No differences in the syntactic categories of the word types emerged.

**Figure 3:**
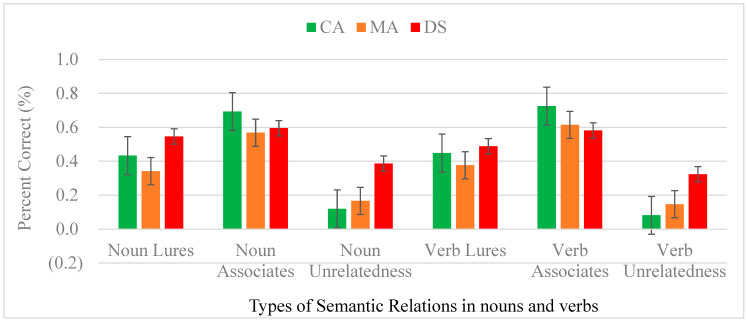
Accuracy of types of semantic relations in nouns and verbs of the three groups. Note: For Noun Lures, the proportion of “yes” responses to lures in noun-eliciting false-memory task; Noun Associates, the proportion of “yes” responses to associates in noun-eliciting false-memory task; Noun Unrelatedness, the proportion of “yes” responses to unrelated words in noun-eliciting false-memory task; Verb Lures, the proportion of “yes” responses to lures in verb-eliciting false-memory task; Verb Associates, the proportion of “yes” responses to associates in verb-eliciting false-memory task; Verb Unrelatedness, the proportion of “yes” responses to unrelated words in verb-eliciting false- memory task.

The main effect of types of words reached significance [*F*(2,114) = 179.553, *p* < 0.001, *ƞ*^2^ = 0.759], suggesting associates received higher “yes” responses (0.630, SE = 0.018) than lures (0.439, SE = 0.023) and unrelated words (0.204, SE = 0.019) at *p* < 0.001. Unrelated words received the least number of responses. The main effect of groups was significant [*F*(2,57) = 5.114, *p* = 0.009, *ƞ*^2^ = 0.152], implying that the DS group pressed the highest frequency of “yes” responses (0.487, SE = 0.026) compared to the CA group (0.417, SE = 0.026) and the MA group (0.369, SE = 0.026). Only the difference between the DS and MA groups was significant (*p* = 0.002). Figure 3 shows the accuracy of word types with error bars across nouns and verbs of the three groups. In sum, people with DS had developmental delays in responding to associates and developmental deviance in processing lures and unrelated words.

**Figure 4:**
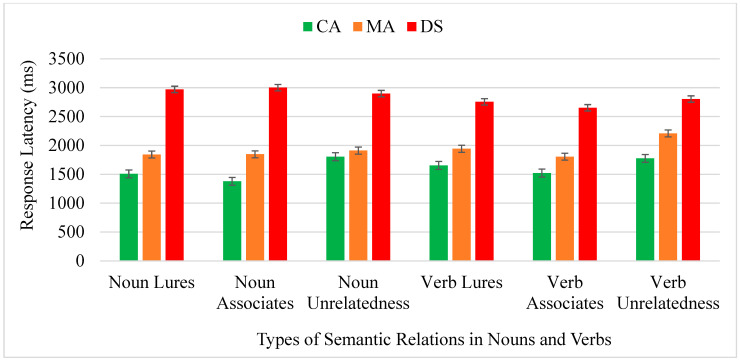
Reaction times of types of semantic relations in nouns and verbs of the three groups. Note: Noun Lures, reaction times of “yes” responses to lures in noun-eliciting false-memory task; Noun Associates, reaction times of “yes” responses to associates in noun-eliciting false-memory task; Noun Unrelatedness, reaction times of “yes” responses to unrelated words in noun-eliciting false-memory task; Verb Lures, reaction times of “yes” responses to lures in verb-eliciting false-memory task; Verb Associates, reaction times of “yes” responses to associates in verb-eliciting false-memory task; Verb Unrelatedness, reaction times of “yes” responses to unrelated words in verb-eliciting false-memory task.

A participant was not counted in the analyses of reaction times because the response time was too long. The three-way ANOVA revealed no interaction. The main effect of word types was significant [*F*(2,82) = 4.500, *p* < 0.014, *ƞ*^2^ = 0.099], suggesting unrelated words were responded to the slowest (2233 ms, SE = 161) compared to the lures (2112 ms, SE = 158) [*p* = 0.030] and the associates (2034 ms, SE = 147) [*p* = 0.026]. The latter two values were not statistically significant. The main effect of groups was significant [*F*(2,46) = 7.117, *p* = 0.002, *ƞ*^2^ = 0.236]. People with DS (2956 ms, SE = 268 ms) were slowest in responding to all trials compared to the two controls (CA, 1588 ms, SE = 277 ms, *p* = 0.001; MA, 1857 ms, SE = 277 ms, *p* = 0.007). No significant differences emerged between the CA and MA groups. Figure 4 shows the reaction times with error bars for the types of nouns and verbs in the three groups.

### 3.3. Analyses of Stimuli Discrimination in Noun-Eliciting False-Memory Task and Verb-Eliciting False-Memory Task

The discrimination sensitivity, i.e., *d*-prime, was calculated and compared between associates from lures and between associates from unrelated words, and these results were analyzed. A one-way ANOVA was employed with *d*-prime values of associates and lures as the within-participants factor and groups as the between-participants factor. The results revealed no differences in the differentiation of associates from lures among groups. Another one-way ANOVA was employed with *d*-prime values of associated and unrelated words as the within-participants factor and groups as the between-participants factor. Significant differences among groups emerged [*F*(2,48) = 7.501, *p* = 0.001, *ƞ*^2^ = 0.238], suggesting the CA group (9.83, SD = 9.37) showed high differentiation of associates from unrelated words compared to the MA group (4.80, SD = 5.78) and the DS group (1.58, SD = 0.713). The latter two groups did not show significant differences. Further analyses of the correlation between participants’ age and the two *d*-prime sensitivity values revealed no significant difference.

The discrimination sensitivity of verbs was put in a one-way ANOVA with the *d*-prime value as a within-participants factor and groups as a between-participants factor. No significant differences emerged between the groups (*F* < 1). The positivity bias differentiating associates from unrelated words yielded significance [*F*(2,46) = 7.710, *p* = 0.001, *ƞ*^2^ = 0.251], implying the DS group (2.18, SD = 1.36) showed the lowest sensitivity value compared with the CA group (9.98, SD = 6.75) and the MA group (8.13, SD = 8.18). There were no significant differences between the two groups. Only the MA group showed a significant correlation between age and sensitivity for differentiating associated from unrelated words [*r* = 0.581, *p* = 0.018]. No significant correlation emerged in the CA and DS groups. This finding suggests that MA controls were sensitive to the differences between associated and unrelated words related to the verb-eliciting false-memory task with age.

Compared to controls, people with DS delayed differentiating associates from unrelated words in the noun-eliciting false-memory task but deviated in differentiating associates from unrelated words in verb-eliciting false-memory tasks. These results were comparable with the hypothesis in the introduction that people with DS would have more difficulty with verbs than nouns.

### 3.4. Analyses of Error Patterns in Noun-Eliciting False-Memory Task

The error pattern was analyzed topic-wise across groups. The nominal topics and word types were the within-participants factors, and groups were the between-participants factor in a three-way analysis of variance. The results revealed a significant three-way interaction [*F*(36,1026) = 1.571, *p* = 0.018, *ƞ*^2^ = 0.052]. One of the simple main effects was from the two-way interaction of topics and groups to lures [*F*(18,513) = 2.273, *p* = 0.002, *ƞ*^2^ = 0.074]. The simple simple main effect was from the group effect on the topics of *public square dancing* [DS, 0.500, SD = 0.296; MA, 0.133, SD = 0.199; CA, 0.116, SD = 0.163; *F*(2,57) = 18.279, *p* < 0.001, *ƞ*^2^ = 0.391], *amusement park* [DS, 0.516, SD = 0.314; MA, 0.266, SD = 0.231; CA, 0.283, SD = 0.311; *F*(2,57) = 4.694, *p* = 0.013, *ƞ*^2^= 0.141], and *summer* [DS, 0.433, SD = 0.326; MA, 0.250, SD = 0.238; CA, 0.216, SD = 0.291; *F*(2,57) = 3.286, *p* = 0.045, *ƞ*^2^ = 0.103]. Based on the topic-wise comparisons, people with DS made more errors than the two control groups with post hoc analyses with the least significant difference at *p* < 0.001 on the topic of *public square dancing* compared to both the CA group and MA group, at *p* = 0.013 (DS vs. CA) and *p* = 0.008 (DS vs. MA) on the topic of *amusement park*, and at *p* = 0.021 (DS vs. CA) and *p* = 0.049 (DS vs. MA) on the topic of *summer*. The remaining group effect was observed on the topics of *breakfast store* [DS, 0.650, SD = 0.366; MA, 0.300, SD = 0.303; CA, 0.500, SD = 0.315; *F*(2,57) = 5.674, *p* = 0.006, *ƞ*^2^= 0.166] and *road* [DS, 0.616, SD = 0.363; MA, 0.300, SD = 0.303; CA, 0.483, SD = 0.295; *F*(2,57) = 4.866, *p* = 0.011, *ƞ*^2^ = 0.146], suggesting higher errors by people with DS than the MA controls on the topics of *breakfast store* (*p* = 0.001) and *road* (*p* = 0.003). Another simple simple main effect was from the topic effect on groups [CA, *F*(9,171) = 9.735, *p* < 0.001, *ƞ*^2^ = 0.339; MA, *F*(9,171) = 7.431, *p* < 0.001, *ƞ*^2^ = 0.281; DS, *F*(9,171) = 2.145, *p* = 0.028, *ƞ*^2^ = 0.101]. All the groups erred highest on the topic of *hospital* (CA, 0.700, SE = 0.064; MA, 0.733, SE = 0.052; DS, 0.667, SE = 0.084); however, the patterns of lowest errors were different in people with DS from the two TD control groups. While the CA and MA groups erred least with *public square dancing* (CA, 0.117, SE = 0.036; MA, 0.133, SE = 0.045), the DS group erred least with *barbershop* (0.433, SE = 0.091) and with *summer* (0.433, SE = 0.073). The main effect of the group reached significance [*F*(2,57) = 5.558, *p* = 0.006, *ƞ*^2^ = 0.163], suggesting the DS group erred more (0.547, SE = 0.044) than the CA group (0.433, SE = 0.044) and the MA group (0.342, SE = 0.044). The difference between the DS and the MA groups was statistically significant (*p* = 0.002).

The interaction of topics and groups in processing associates and unrelated words failed to reach significance. The main effect of groups in processing associates was significant [*F*(2,57) = 3.631, *p* = 0.033, *ƞ*^2^ = 0.113], suggesting the CA group erred least (0.307, SE = 0.035) compared with the MA group (0.432, SE = 0.035, *p* = 0.013) and the DS group (0.405, SE = 0.035, *p* = 0.049). The lowest errors emerged with *ho*spital** (0.217, SE = 0.039); the highest was with *summer* (0.472, SE = 0.038). Moreover, the main effect of the group in processing the unrelated words was significant [*F*(2,57) = 16.733, *p* < 0.001, *ƞ*^2^ = 0.370], suggesting the DS group erred most (0.387, SE = 0.035) compared with the CA group (0.120, SE = 0.035) and the MA group (0.167, SE = 0.035) at *p* < 0.001. In sum, people with DS made errors in associates like the MA controls (i.e., delay), but they made more errors in unrelated words the most (i.e., deviance). The error patterns of people with DS differed from those of the two control groups.

Another simple main effect was the two-way interaction between the type and group for each topic. The results showed the topics of the *Dragon Boat Festival*, *public square dancing*, *bathroom*, *road*, and *summer* reached significance. Table 2 presents the statistical results.

**Figure 5:**
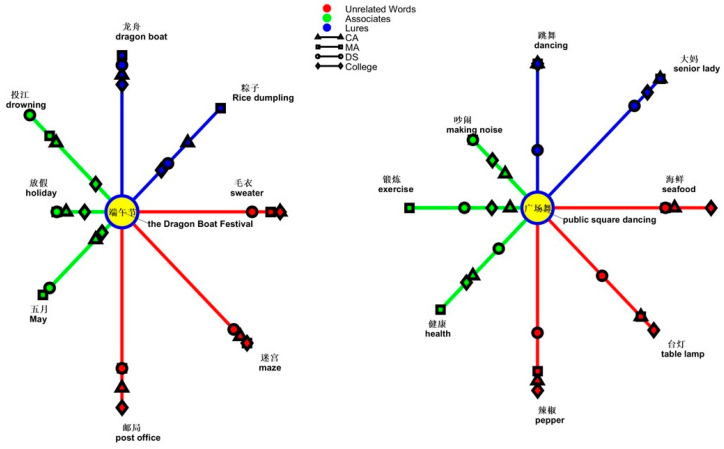
Semantic relatedness of the four groups in the nominal topics of the *Dragon Boat Festival* and *public square dancing*.

In the analysis of the topic of the *Dragon Boat Festival*, the simple main effect was from group effect on associates [*F*(2,57) = 3.485, *p* = 0.037, *ƞ*^2^ = 0.109]. The DS group erred more (0.533, SD = 0.199) than the MA group (0.500, SD = 0.253, *p* = 0.017), which was greater than that of the CA group (0.333, SD = 0.305, *p* = 0.045). No differences were observed between the groups for lures or unrelated words. The other simple main effect was from types of words on groups [CA, *F*(2,38) = 7.858, *p* = 0.001, *ƞ*^2^ = 0.293; MA, *F*(2,38) = 8.703, *p* = 0.001, *ƞ*^2^ = 0.314; DS, *F*(2,38) = 10.916, *p* < 0.001, *ƞ*^2^ = 0.365]. People with DS erred least with unrelated words (0.217, SE = 0.065) than with lures (0.517, SE = 0.074) and associates (0.533, SE = 0.045) at *p* = 0.001. The patterns observed in the CA group were similar to those in people with DS (unrelated words, 0.117, SE = 0.050; associates, 0.333, SE = 0.068; lures, 0.517, SE = 0.085). The difference between unrelated words and lures was significant (*p* = 0.001), as was the difference between unrelated words and associates (*p* = 0.008). However, the MA group made more errors with associates (0.500, SE = 0.057) than lures (0.300, SE = 0.072) and unrelated words (0.150, SE = 0.051). The difference between associates and lures was *p* = 0.019, and the difference between associates and unrelated words was *p* < 0.001. The results revealed different patterns in the MA group compared to the CA and DS groups. A graph shows “yes” responses as the semantic distance between the topic of the *Dragon Boat Festival* and the types of words in Figure 5. The data of college students are included in the graph to uncover the whole picture of semantic relatedness.

In the analysis of the topic of *public square dancing*, the interaction of types of words and groups reached significance [*F*(4,114) = 6.755, *p* < 0.001, *ƞ*^2^ = 0.192]. The simple main effect was from the group effect on lures [*F*(2,57) = 5.674, *p* = 0.006, *ƞ*^2^ = 0.116] and unrelated words [*F*(2,57) = 3.572, *p* = 0.035, *ƞ*^2^ = 0.111]. People with DS made more errors (0.650, SE = 0.074) with lures than did the CA (0.500, SE = 0.074) and MA groups (0.300, SE = 0.074). Only the difference of people with DS and the MA group was significant (*p* = 0.001). People with DS (0.450, SE = 0.066) erred more than the two control groups (0.233, SE = 0.066) with unrelated words, suggesting deviant processing in people with DS. Another simple main effect was from the effect of word types on control groups [CA, *F*(2,38) = 4.815, *p* = 0.014, *ƞ*^2^ = 0.202; MA, *F*(2,38) = 19.046, *p* < 0.001, *ƞ*^2^ = 0.501; DS, *F* < 1]. In the CA and MA groups, associates received more errors than lures (CA, *p* = 0.012; MA, *p* < 0.001) and unrelated words (CA, *p* = 0.049; MA, *p* = 0.001) [CA, associates, 0.300, SD = 0.239; lures, 0.116, SD = 0.163; unrelated words, 0.183, SD = 0.253; MA, associates, 0.650, SD = 0.275; lures, 0.133, SD = 0.199; unrelated words, 0.216, SD = 0.311]. However, people with DS did not show this pattern. Instead, people with DS erred more with lures (0.500, SE = 0.066) than with associates (0.400, SE = 0.071) or unrelated words (0.383, SE = 0.070). The latter two types of words yielded no difference. This finding suggests that people with DS were deviant in making the highest “yes” responses to unrelated words and showed a distinct pattern of higher errors with lures than the control groups. A graph shows “yes” responses as the semantic distance between the topic of *public square dancing* and the types of words in Figure 5.

**Figure 6:**
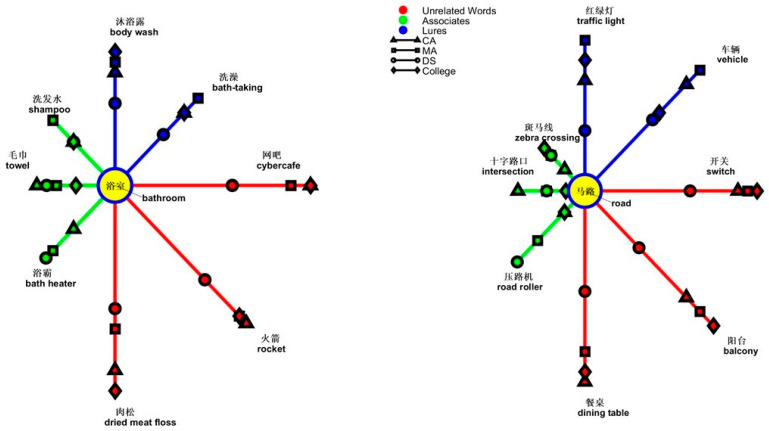
Semantic relatedness of the four groups in the nominal topics of *Bathroom* and *Road*.

In the analysis of the topic of *bathroom*, the interaction of types of words and groups reached significance [*F*(4,114) = 2.731, *p* = 0.032, *ƞ*^2^ = 0.087]. The simple main effect was from the group effect on unrelated words [*F*(2,57) = 7.320, *p* = 0.001, *ƞ*^2^ = 0.204], suggesting people with DS made more errors than the CA group (*p* < 0.001) and the MA group (*p* = 0.017). Another simple main effect was from types of words in groups [CA, *F*(2,38) = 23.413, *p* < 0.001, *ƞ*^2^ = 0.552; MA, *F*(2,38) = 3.973, *p* = 0.027, *ƞ*^2^ = 0.173]. However, this difference was not significant in the DS group. In the CA and MA groups, lures received more errors than associates or unrelated words (CA, lures, 0.600, SD = 0.255; associates, 0.333, SD = 0.305; unrelated words, 0.050, SD = 0.122; MA, lures, 0.416, SD = 0.322; associates, 0.400, SD = 0.298; unrelated words, 0.166, SD = 0.315). The CA group showed a difference between unrelated words and lures at *p* < 0.001 and a difference between unrelated words and associates at *p* = 0.001. The difference between lures and associates was *p* = 0.012. The MA group showed a difference between unrelated words and lures at *p* = 0.010 and a difference between unrelated words and associates at *p* = 0.019. The MA group did not show a difference between lures and associates. A graph shows “yes” responses as the semantic distance between the topic of *bathroom* and the types of words in Figure 6.

In the analysis of the topic of *road*, the interaction of types of words and groups was significant [*F*(4,114) = 2.721, *p* = 0.033, *ƞ*^2^ = 0.087]. The simple main effect was from the group effect on lures [*F*(2,57) = 4.866, *p* = 0.011, *ƞ*^2^ = 0.146] and unrelated words [*F*(2,57) = 10.756, *p* < 0.001, *ƞ*^2^ = 0.274]. People with DS made more errors with lures than those in the MA group (*p* = 0.003). People with DS erred more with unrelated words than the MA and CA groups, both at *p* < 0.001. Another simple main effect was from differences among types of words in the CA group [*F*(2,38) = 8.412, *p* = 0.001, *ƞ*^2^ = 0.307] and the DS group [*F*(2,38) = 5.159, *p* = 0.010, *ƞ*^2^ = 0.214]. In the CA group, lures (0.483, SD = 0.295) received more errors than associates (0.216, SD = 0.223, *p* = 0.014) and unrelated words (0.166, SD = 0.299, *p* = 0.001). In the DS group, lures (0.616, SD = 0.363) received more errors than unrelated words (0.516, SD = 0.350) or associates (0.316, SD = 0.275), both at *p* = 0.012. A graph shows “yes” responses as the semantic distance between the topic of *road* and the types of words in Figure 6.

**Figure 7:**
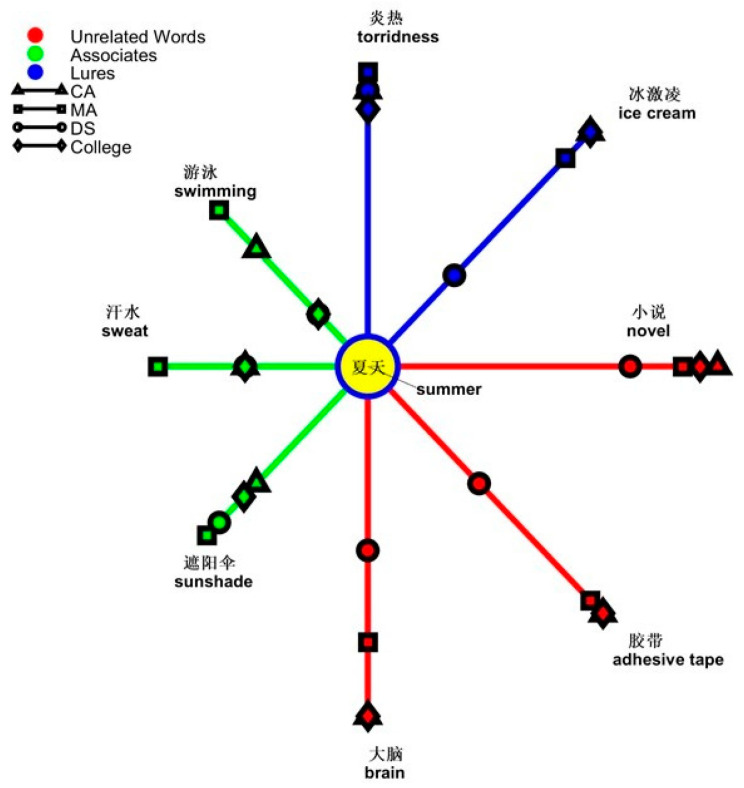
Semantic relatedness of the four groups in the nominal topic of *Summer*.

In the analysis of the topic of *summer*, the interaction of types of words and groups was significant [*F*(4,114) = 5.919, *p* < 0.001, *ƞ*^2^ = 0.172]. The simple main effect was from group effect on types of words [lures, *F*(2,57) = 3.286, *p* = 0.045, *ƞ*^2^ = 0.103; associates, *F*(2,57) = 3.643, *p* = 0.032, *ƞ*^2^ = 0.113; unrelated words, *F*(2,57) = 17.401, *p* < 0.001, *ƞ*^2^ = 0.379]. Regarding the error patterns of lures and unrelated words, the DS group made more errors than the CA and MA groups (lures, DS, 0.433, SE = 0.064; CA, 0.217, SE = 0.064; MA, 0.250, SE = 0.064; unrelated words, DS, 0.433, SE = 0.049; CA, 0.033, SE = 0.049; MA, 0.150, SE = 0.049). For lures, the difference between the DS group and the MA group was at *p* = 0.049, and the difference between the DS group and the CA group was at *p* = 0.021. In unrelated words, the difference between the DS group and two control groups was *p* < 0.001. However, in the error pattern of associates, the MA group (0.617, SE = 0.066) erred most compared to the CA group (0.417, SE = 0.066, *p* = 0.037) and DS group (0.383, SE = 0.066, *p* = 0.015). Another simple main effect was from differences among types of words in the CA group [*F*(2,38) = 12.938, *p* < 0.001, *ƞ*^2^ = 0.405] and the MA group [*F*(2,38) = 15.721, *p* < 0.001, *ƞ*^2^ = 0.453]. Both the CA and MA groups erred more with associates (CA: 0.417, SE = 0.063; MA: 0.617, SE = 0.078) than with lures (CA: 0.217, SE = 0.065; MA: 0.250, SE = 0.053) or unrelated words (CA: 0.033, SE = 0.023; MA: 0.150, SE = 0.045). Together, these results suggest that people with DS show deviations in processing summer-related semantic words. A graph shows “yes” responses as the semantic distance between the topic of *summer* and the types of words in Figure 7.

No interaction emerged among the processing of *barbershop* and *breakfast store*, *amusement park*, *Christmas*, and *hospital*. People with DS erred most compared with the CA and MA controls. Significant differences among the groups emerged in processing *barbershop*, *breakfast store*, *public square dancing*, *amusement park*, *bathroom*, *road*, and *summer*. The topic-wise analyses yielded effects on word types in each group. In the CA group, all the effects of the types of words were significant (except for the topic of *amusement park*). In the MA group, almost all effects of the types of words were significant (except for *breakfast store*, *amusement park*, and *road*). In the DS group, almost all effects of the types of words were not significant (except for the *Dragon Boat Festival*, *road*, and *hospital*). These findings suggest that people with DS deviate from forming concepts related to nouns through lexical semantics.

### 3.5. Analyses of Error Patterns in Verb-Eliciting False-Memory Task

To determine which semantic concepts of verbs were fragile in people with DS compared to controls, the error patterns of topics in each group were analyzed. Verbal topics and types of words were taken as the within-participants factors and groups as the between-participants factor in a three-way analysis of variance. The results revealed a significant three-way interaction [*F*(36,1026) = 2.083, *p* < 0.001, *ƞ*^2^ = 0.068]. One of the simple main effects was from the two-way interaction of topics and groups in processing lures [*F*(18,513) = 2.566, *p* < 0.001, *ƞ*^2^ = 0.083]. The simple simple main effect was from the group effect on *do housework* [*F*(2,57) = 3.243, *p* = 0.046, *ƞ*^2^ = 0.102], *transport during Spring Festival* [*F*(2,57) = 3.917, *p* = 0.025, *ƞ*^2^ = 0.121], *get married* [*F*(2,57) = 3.966, *p* = 0.024, *ƞ*^2^ = 0.122], and *shop online*. In the processing of the former two topics and the last one, the DS group erred more than the two control groups [*do housework*, DS, 0.583, SD = 0.402; CA, 0.533, SD = 0.380; MA, 0.300, SD = 0.340; *transport during Spring Festival*, DS, 0.466, SD = 0.294; CA, 0.283, SD = 0.311; MA, 0.216, SD = 0.270; *shop online*, DS, 0.650, SD = 0.350; CA, 0.500, SD = 0.275; MA, 0.350, SD = 0.253]. Only the differences between the DS group and MA group were significant (*do housework*, *p* = 0.020; *transport during the Spring Festival*, *p* = 0.009; *shop online*, *p* = 0.002). When processing the concept of *get married*, the DS group (0.466, SD = 0.313) produced fewer “yes” responses compared with the two control groups (0.716, SD = 0.329). The differences between the DS group and the two controls were significant (*p* = 0.018).

Another simple simple main effect was from the effect of types of words on groups [CA, *F*(9,171) = 5.018, *p* < 0.001, *ƞ*^2^ = 0.209; MA, *F*(9,171) = 5.373, *p* < 0.001, *ƞ*^2^ = 0.220; DS, *F*(9,171) = 2.996, *p* = 0.002, *ƞ*^2^ = 0.136]. The error patterns differed between the DS and control groups. In the two control groups, both groups erred most with *get married* (0.716, SD = 0.329) and least with *celebrate birthday* (CA, 0.250, SD = 0.283; MA, 0.216, SD = 0.270) and *transport during Spring Festival* (CA, 0.283, SD = 0.311; MA, 0.216, SD = 0.270). However, people with DS erred most with *shop online* (0.650, SD = 0.350) and least with *celebrate birthday* (0.400, SD = 0.368) and *play the piano* (0.283, SD = 0.369).

Another simple main effect was from the effect of types of words on processing associates [*F*(18,513) = 2.003, *p* = 0.008, *ƞ*^2^ = 0.066]. The simple simple main effect was from the group effect on *get married* [*F*(2,57) = 10.121, *p* < 0.001, *ƞ*^2^ = 0.262], *celebrate New Year* [*F*(2,57) = 5.894, *p* = 0.005, *ƞ*^2^ = 0.171], and *play the piano* [*F*(2,57) = 4.101, *p* = 0.022, *ƞ*^2^ = 0.126]. In the processing of the former two topics, people with DS erred most among groups [*get married*, DS, 0.550, SD = 0.329; MA, 0.283, SD = 0.291; CA, 0.150, SD = 0.228; *celebrate New Year*, DS, 0.366, SD = 0.284; MA, 0.250, SD = 0.303; CA, 0.083, SD = 0.183]. In the processing of *play the piano*, people with DS (0.416, SD = 0.322) made errors similar to those in the CA group (0.383, SD = 0.311), which were fewer than those in the MA group (0.633, SD = 0.262). Another simple simple main effect was from the effect of topics on groups [CA, *F*(9,171) = 4.400, *p* < 0.001, *ƞ*^2^ = 0.188; MA, *F*(9.171) = 4.812, *p* < 0.001, *ƞ*^2^ = 0.202]. No significant differences were observed between the DS and two control groups. Moreover, the error patterns differed between the CA and MA groups. In the CA group, the errors with *transport during Spring Festival* were the highest (0.400, SD = 0.352) and the errors with *celebrate the New Year* (0.083, SD = 0.183) and *get married* (0.150, SD = 0.228) were the least. In the MA group, the errors with *play the piano* were the highest (0.633, SD = 0.262) and the errors with *celebrate New Year* (0.250, SD = 0.303) and *watch movies* (0.250, SD = 0.262) were the least. The interaction between topics and groups in processing unrelated words was not significant. People with DS erred most among groups with the unrelated words [main effect of group, *F*(2,57) = 13.132, *p* < 0.001, *ƞ*^2^ = 0.315; DS, 0.323, SE = 0.035; MA, 0.147, SE = 0.035; CA, 0.082, SE = 0.035].

In sum, people with DS were deviant in responding to lures of the verb-eliciting topics of *do housework*, *transport during Spring Festival*, *get married*, and *shop online*. People with DS showed deviant error patterns in processing lures that differed from the control groups. People with DS were also deviant in responding to associates of the verb-eliciting topics *get married*, *celebrate New Year*, and *play the piano*. Unlike the two control groups, people with DS showed similar “yes” responses to all topics, without significant differences for any specific topic in the processing of associates. To clarify the nature of processing verb-eliciting topics in false-memory tasks across groups, topic-based statistical results are listed in Table 3.

In the processing of *do housework*, the interaction of types and groups reached significance [*F*(4,114) = 2.778, *p* = 0.030, *ƞ*^2^ = 0.089]. The simple main effect was from group effect on types [lures, *F*(2,57) = 3.243, *p* = 0.046, *ƞ*^2^ = 0.102; unrelated words, *F*(2,57) = 5.778, *p* = 0.005, *ƞ*^2^ = 0.169]. In the processing of lures, people with DS (0.583, SD = 0.402) erred more than those with CA (0.533, SD = 0.380) or MA (0.300, SD = 0.340). The difference between the DS group and the MA group was at *p* = 0.020. In the processing of unrelated words, people with DS (0.250, SD = 0.283) erred more than those with CA (0.016, SD = 0.074) or MA (0.183, SD = 0.253). The difference between the DS group and the CA group was at *p* = 0.002; the difference between the CA and MA group was at *p* = 0.022.

**Figure 8:**
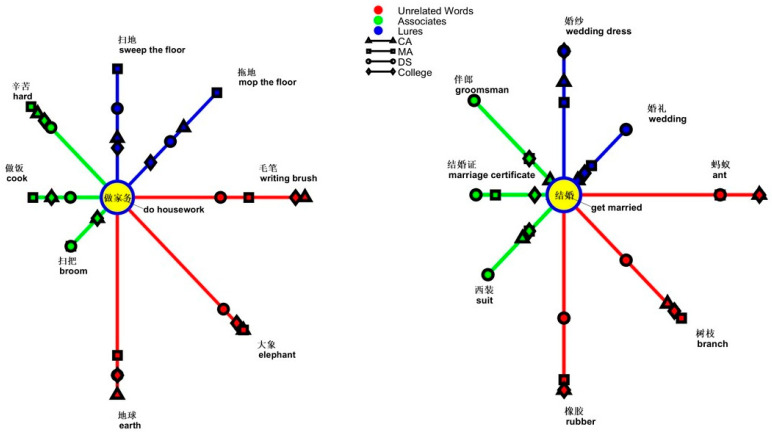
Semantic relatedness of the four groups in the verbal topics of *do housework* and *get married*.

Another simple main effect was from the effect of types of words on groups [CA, *F*(2,38) = 13.103, *p* < 0.001, *ƞ*^2^ = 0.408; MA, *F*(2,38) = 4.217, *p* = 0.022, *ƞ*^2^ = 0.182; CA, *F*(2,38) = 3.690, *p* = 0.034, *ƞ*^2^ = 0.163]. People with DS responded more to lures than to associates or unrelated words (CA, lures, 0.533, SE = 0.085; associates, 0.367, SE = 0.068; unrelated words, 0.017, SE = 0.017; DS, lures, 0.583, SE = 0.090; associates, 0.367, SE = 0.072; unrelated words, 0.250, SE = 0.063). The differences between lures and associates or lures and unrelated words in the CA group were both at *p* < 0.001. The difference between lures and unrelated words in the DS group was at *p* = 0.003. However, the MA group responded more strongly to associates (0.483, SD = 0.295) than to lures (0.300, SD = 0.340) or unrelated words (0.183, SD = 0.253). A graph showing “yes” responses as the semantic distance between the topic of *do housework* and the types of words is shown in Figure 8.

In the processing of *get married*, the interaction of types and groups reached significance [*F*(4,114) = 7.658, *p* < 0.001, *ƞ*^2^ = 0.212]. The simple main effect was from group effect on types [lures, *F*(2,57) = 3.966, *p* = 0.024, *ƞ*^2^ = 0.122; associates, *F*(2,57) = 10.121, *p* < 0.001, *ƞ*^2^ = 0.262; unrelated words, *F*(2,57) = 8.471, *p* = 0.001, *ƞ*^2^ = 0.229]. People with DS deviated from processing topics. When processing associates and unrelated words, most people with DS erred most significantly among groups (associates, DS: 0.550, SD = 0.329; MA: 0.283, SD = 0.291; CA: 0.150, SD = 0.228; unrelated words: DS, 0.383, SD = 0.291; MA, 0.150, SD = 0.228; CA, 0.100, SD = 0.156). In the processing of lures, people with DS had significantly fewer “yes” responses than those in the two control groups (DS, 0.466, SD = 0.313; MA, CA, 0.717, SD = 0.329). Another simple main effect was from the effect of types of words on groups [CA, *F*(2,38) = 39.271, *p* < 0.001, *ƞ*^2^ = 0.674; MA, *F*(2,38) = 20.193, *p* < 0.001, *ƞ*^2^ = 0.515]. No difference was observed in the DS group. Both the CA and MA groups erred more with lures than with associates and unrelated words. The graph showing “yes” responses as the semantic distance between the topic of *get married* and types of words is depicted in Figure 8.

**Figure 9:**
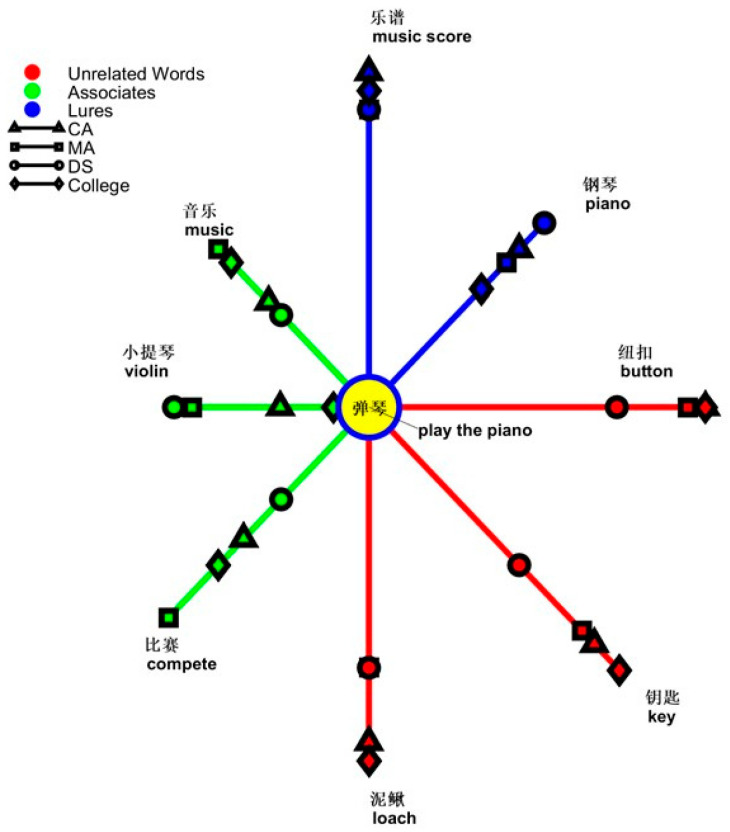
Semantic relatedness of the four groups in the verbal topic of *play the piano*.

In the processing of *play the piano*, the interaction of types and groups reached significance [*F*(4,114) = 2.563, *p* = 0.042, *ƞ*^2^ = 0.083]. The simple main effect was from the group effect on types of words [associates, *F*(2,57) = 4.101, *p* = 0.022, *ƞ*^2^ = 0.126; unrelated words, *F*(2,57) = 4.682, *p* = 0.013, *ƞ*^2^ = 0.141]. People with DS erred more with unrelated words than those in the MA and CA groups (DS, 0.333, SD = 0.324; MA, 0.183, SD = 0.253; CA, 0.083, SD = 0.183). Only the difference between the DS group and the CA group was statistically significant (*p* = 0.004). People with DS did not show significant differences in the error patterns associated with the CA group (DS, 0.416, SD = 0.322; CA, 0.383, SD = 0.311), which were significantly lower than those in the MA group (0.633, SD = 0.262). No difference was observed in lure processing among the groups, although the MA group (0.350, SD = 0.295) responded more positively than the CA (0.283, SD = 0.311) and DS (0.283, SD = 0.329) groups. Another simple main effect was from the effect of types of words on groups [CA, *F*(2,38) = 8.430, *p* = 0.001, *ƞ*^2^ = 0.307; MA, *F*(2,38) = 15.596, *p* < 0.001, *ƞ*^2^ = 0.451]. Both control groups responded more accurately to associates than to lures or unrelated words. In the CA group, the difference between unrelated words and associates was at *p* = 0.001; the difference between unrelated words and lures was at *p* = 0.010. In the MA group, the difference between unrelated words and associates was at *p* < 0.001; the difference between unrelated words and lures was at *p* = 0.029. The difference between the associates and lures was at *p* = 0.009. No differences in word types were observed in the DS group. The graph showing “yes” responses as the semantic distance between the topic of *play the piano* and types of words is depicted in Figure 9.

Taken together, people with DS deviated in processing *do housework*, *get married*, and *play the piano*. Moreover, people with DS erred most among groups in the processing of *transport during Spring Festival*, *celebrate New Year*, *watch movies*, and *shop online* [main effect of group, *transport during Spring Festival*, *F*(2,57) = 5.780, *p* = 0.005, *ƞ*^2^ = 0.169; *celebrate New Year*, *F*(2,57) = 6.559, *p* = 0.003, *ƞ*^2^ = 0.187; *watch movies*, *F*(2,57) = 11.807, *p* < 0.001, *ƞ*^2^ = 0.293; *shop online*, *F*(2,57) = 11.522, *p* < 0.001, *ƞ*^2^ = 0.288]. People with DS did not show group differences in the processing of *celebrate birthday*, *raise flag*, and *get lost*.

Another simple main effect was from the interaction of topics and types of words in groups [CA, *F*(18,380) = 4.303, *p* < 0.001, *ƞ*^2^ = 0.169; MA, *F*(18,380) = 3.909, *p* < 0.001, *ƞ*^2^ = 0.156]. No interaction emerged in people with DS. The main effect of types of words on the three groups reached significance [CA, *F*(2,380) = 100.056, *p* < 0.001, *ƞ*^2^ = 0.345; MA, *F*(2,380) = 45.850, *p* < 0.001, *ƞ*^2^ = 0.194; DS, *F*(2,380) = 12.568, *p* < 0.001, *ƞ*^2^ = 0.062]. People in the DS and the CA group responded more accurately to lures (CA: 0.448, SE = 0.023; DS: 0.488, SE = 0.025) than to associates (CA: 0.275, SE = 0.020; DS: 0.418, SE = 0.021) or unrelated words (CA: 0.082, SE = 0.012; DS: 0.323, SE = 0.022). The MA group responded more accurately to associated words (0.385, SE = 0.021) than to lures (0.377, SE = 0.022) or unrelated words (0.147, SE = 0.016). In the CA and MA groups, the main effect of topics was significant [CA, *F*(9,190) = 2.155, *p* = 0.027, *ƞ*^2^ = 0.093; MA, *F*(9,190) = 2.279, *p* = 0.019, *ƞ*^2^ = 0.097]. Error patterns in the CA and MA groups were similar. Three topics received the highest errors for associates, and over half received the highest errors for lures. The topic of *get lost* was not significant. People with DS show deviant error patterns in verb-eliciting false-memory tasks.

Regression analyses of dependent variables with the standardized tests of WSIC were employed with working memory forward digit span length and score, working memory backward digit span length and score, and total memory length and score. The results revealed that total memory length negatively predicted the yes/response proportion of unrelated words in verbs for people with DS (*r* = −0.455, adjusted R^2^ = 0.163, *F*(1,19) = 4.706, *p* = 0.04). This finding suggests that people with DS might use a verbatim strategy to memorize the stimuli but not automatically integrate the words into semantic memory. No other factor emerged as a predictor of accuracy or reaction times for any group of nouns or verbs in this study.

## 4. Discussion

This study used the classic false-memory paradigm to investigate the conceptual formation of semantic knowledge of people with DS. By presenting semantically related words in the study phase and mixing semantically unrelated words with conceptually integrated lures in the recognition phase, “yes” responses to each word type were calculated. Data from college students were taken as the baseline to examine the impact of syntactic categories on word types in false-memory tasks. No interaction of syntactic categories and word types emerged, suggesting no difference in syntactic categories in recognition accuracy. The associates were responded to most accurately among word types. The unrelated words received fewer “yes” responses. However, differences in syntactic categories in response latencies emerged, suggesting responses in the noun-eliciting false-memory task were faster than the ones in the verb-eliciting false-memory. These were the baseline for comparison of people with DS and the TD controls. In comparisons of the three groups, people with DS made “yes” responses to all types of words more than the TD controls. People with DS showed delayed processing of associates and deviant processing of lures and unrelated words. Asymmetrical processing of nouns and verbs has emerged in the discrimination of word types in people with DS. In the noun-eliciting false-memory task, people with DS showed a delay in differentiating associates from unrelated words; in the verb-eliciting false-memory task, people with DS showed deviance in differentiating associates from unrelated words. More evidence of problematic semantic processing of words in people with DS compared to the control groups emerged, indicating the atypical development of semantic networks in people with DS in conceptual integration from semantic relatedness. The atypical patterns emerged in people with DS by showing the lowest accuracy for all types of words compared to the control groups, significantly in certain concepts with nouns (the *Dragon Boat Festival*, *public square dancing*, *bathroom*, *road*, and *summer*) and verbs (*do housework*, *get married*, and *play the piano*). Similar to Hsu [6,7], the findings in our study showed through the use of the false-memory task that people with DS were deviant in semantic processing, as shown in the current study.

The false-memory task was developed by Roediger and McDermott [4] as the classic paradigm in probing false memory, which was similar to virtual sentential processing based on semantically related words that formed semantic networks. The ability of sentential processing is the foundation of social cognition. If people with DS have difficulties in sentential processing, their social cognition is impaired. If people with DS have difficulties in lexical words, their comprehension ability of sentences is deficient. Hence, the semantic knowledge of lexical words of people with DS is the root cause of probing their semantic organization and social cognition. Based on our findings, people with DS were atypical in their comprehension of lexical semantics. The impairment of lexical semantics in people with DS might result from bizarre semantic features of words in their semantic network.

Based on the spreading activation model [11], a word is a concept node that interacts with other nodes. These concepts are grouped closer together because of their shared semantic features. Our analyses of the error patterns for each topic in people with DS revealed possible bizarre semantic features embedded in lexical semantics, leading to atypical semantic knowledge. This hypothesis of bizarre semantic features of lexical semantics is consistent with the hypotheses of dosage imbalance and amplified developmental instability in people with DS related to genetic impairment in the early stage. This impairment results in atypical neurodevelopment and neurodegeneration in people with DS [12].

Anderson [13] considered a node a cognitive unit with connected elements or semantic features. Each unit is processed in the working memory and spread to other units in the long-term memory with the impact of connection strength. Whenever a word is processed, it undergoes an encoding stage, which creates a trace with different strengths and which influences retrieval speed. The second stage involves the storage or retention of words. The trace is not lost but decays with time. The last stage is retrieval, in which spreading activation occurs, and levels of processing play a role. Based on this analysis, people with DS may show impairment at any stage, which leads to deviant recognition in false-memory tasks. Furthermore, nominal and verbal lures result in distinct processing patterns in people with DS compared to typically developing controls.

The connectionist model proposed by Rumelhart, Hinton, and McClelland [14] adopts a parallel-distributed processing perspective to account for human information processing. There are input units that carry integrated information, hidden units that are invisible but determine each type of representation, and output units that perform computations to realize connectivity among the units. For people with DS, impairments can occur at the inputs, hidden units, or outputs. Any impairment results in a breakdown of connectivity within the representation system. It may account for deficits in semantic processing and atypical semantic organization in people with DS.

## 5. Conclusions

Impaired processing of lexical semantics might be distinct at behavioral and neurological levels. A study using the false-memory paradigm to investigate the integration ability of people with WS confirmed this asymmetry [15]. While people with WS showed typical behavior, they showed atypical neurological processing. They processed semantic-related but nonpresented lures as the displayed words. Further, they simultaneously showed delayed and deviant semantic organization at the processing stage. It was unclear whether people with DS would show brain and behavioral asymmetry while processing semantically related words. Future studies should be conducted with neuroimaging techniques to investigate lexical semantics in people with DS. Future interventions for people with DS should focus on building up the concept of semantic knowledge, which is highly related to their social cognition. This study could serve as the foundation for interventions for people with DS to enrich their knowledge of lexical semantics; hence, people with DS could enhance their language ability and social cognition. It is the practical implication for the research field of people with DS.

## Figures and Tables

**Table 1:** Background information of participants.

Tasks	Groups	N (F:M)	CA	CA Range (SD)	MA	MA Range (SD)
Category-based false-memory tasks	CA	20 (9:11)	17.7	9.9–22.7 (3.9)	---	---
	MA	20 (9:11)	9.4	4.5–14.7 (3.0)	---	---
	DS	20 (9:11)	17.7	9.4–22.10 (3.9)	9.4	4.4–14.5 (3.0)
	College students	20 (10:10)	23.5	18.8–26.11 (2.4)	---	---
Generating task	College students for nouns	20 (10:10)	22.6	18.7–27.9 (2.6)	---	---
	College students for verbs	20 (10:10)	21.4	18.1–31.1 (3.5)	---	---
Rating task	College students for nouns	20 (10:10)	22.0	19.0–26.11 (2.7)	---	---
	College students for verbs	20 (10:10)	22.8	19.0–26.7 (2.4)	---	---

Note. CA, chronological age-matched controls; MA, mental age-matched controls; DS, people with Down syndrome; SD, standard deviation; F, female; M, male.

**Table 2:** Error analyses of types of words under each nominal topic of the three groups.

Topic	Topic Chinese Name	Topic English Name	Topic × Group Interaction ^a^	Main Effect of Type under Topic ^b^	Main Effect of Group under Topic ^c^
1	理发店	barbershop	2.126, *p* = 0.082	10.901, *p* < 0.001	5.029, *p* = 0.01
2	端午节	Dragon Boat Festival	2.484, *p* = 0.048	21.693, *p* < 0.001	2.239, *p* = 0.116
3	早餐店	breakfast store	1.743, *p* = 0.145	5.909, *p* = 0.004	7.576, *p* = 0.001
4	广场舞	public square dancing	6.755, *p* < 0.001	9.916, *p* < 0.001	12.63, *p* < 0.001
5	游乐园	amusement park	0.812, *p* = 0.52	1.989, *p* = 0.142	10.32, *p* < 0.001
6	浴室	bathroom	2.731, *p* = 0.032	19.055, *p* < 0.001	4.754, *p* = 0.012
7	马路	road	2.721, *p* = 0.033	9.962, *p* < 0.001	9.401, *p* < 0.001
8	夏天	summer	5.919, *p* < 0.001	15.059, *p* < 0.001	7.848, *p* < 0.001
9	圣诞节	Christmas	1.033, *p* = 0.393	22.652, *p* < 0.001	2.313, *p* = 0.108
10	医院	hospital	1.447, *p* = 0.223	51.097, *p* < 0.001	2.443, *p* = 0.096

Note: ^a^ *F*_(4,114)_ for topic × group interaction, ^b^ *F*_(2,114)_ for main effect of type under topic, ^c^ *F*_(2,57)_ for main effect of group under topic.

**Table 3:** Error analyses of types of words under each verbal topic of the three groups.

Topic	Topic Chinese Name	Topic English Name	Topic × Group Interaction ^a^	Main Effect of Type under Topic ^b^	Main Effect of Group under Topic ^c^
1	庆生	celebrate birthday	1.3, *p* = 0.274	8.988, *p* < 0.001	2.663, *p* = 0.078
2	做家务	do housework	2.778, *p* = 0.03	14.086, *p* < 0.001	3.888, *p* = 0.026
3	春运	transport during the Spring Festival	0.332, *p* = 0.856	19.087, *p* < 0.001	5.78, *p* = 0.005
4	结婚	get married	7.658, *p* < 0.001	31.469, *p* < 0.001	5.279, *p* = 0.008.
5	升旗	raise a flag	2.256, *p* = 0.067	16.978, *p* < 0.001	0.8, *p* = 0.454
6	过年	celebrate New Year	2.21, *p* = 0.072	28.045, *p* < 0.001	6.559, *p* = 0.003
7	迷路	get lost	1.266, *p* = 0.288	6.505, *p* = 0.002	2.302, *p* = 0.109
8	看电影	watch movies	0.274, *p* = 0.894	16.719, *p* < 0.001	11.807, *p* < 0.001
9	弹琴	play the piano	2.563, *p* = 0.042	14.641, *p* < 0.001	3.214, *p* = 0.048
10	网购	shop online	2.179, *p* = 0.076	16.834, *p* < 0.001	11.522, *p* < 0.001

Note. ^a^ *F*_(4,114)_ for topic × group interaction, ^b^ *F*_(2,114)_ for main effect of type under topic, ^c^ *F*_(2,57)_ for main effect of group under topic.

## Data Availability

The data presented in this study are available on request from the corresponding author. The data are not publicly available because of ethical concerns.
